# Supplementary material for: Capturing the inherent structural dynamics of the HIV-1 envelope glycoprotein fusion peptide
Source: Nat Commun. 2019 Feb 15;10:763. doi: 10.1038/s41467-019-08738-5 (PMC6377653; doi:10.1038/s41467-019-08738-5)
Supplement: Supplementary file 1 — Supplementary Information [file 41467_2019_8738_MOESM1_ESM.pdf]

Supplementary Information for

Capturing the inherent structural dynamics of the HIV-1 envelope glycoprotein fusion peptide

Kumar et al.

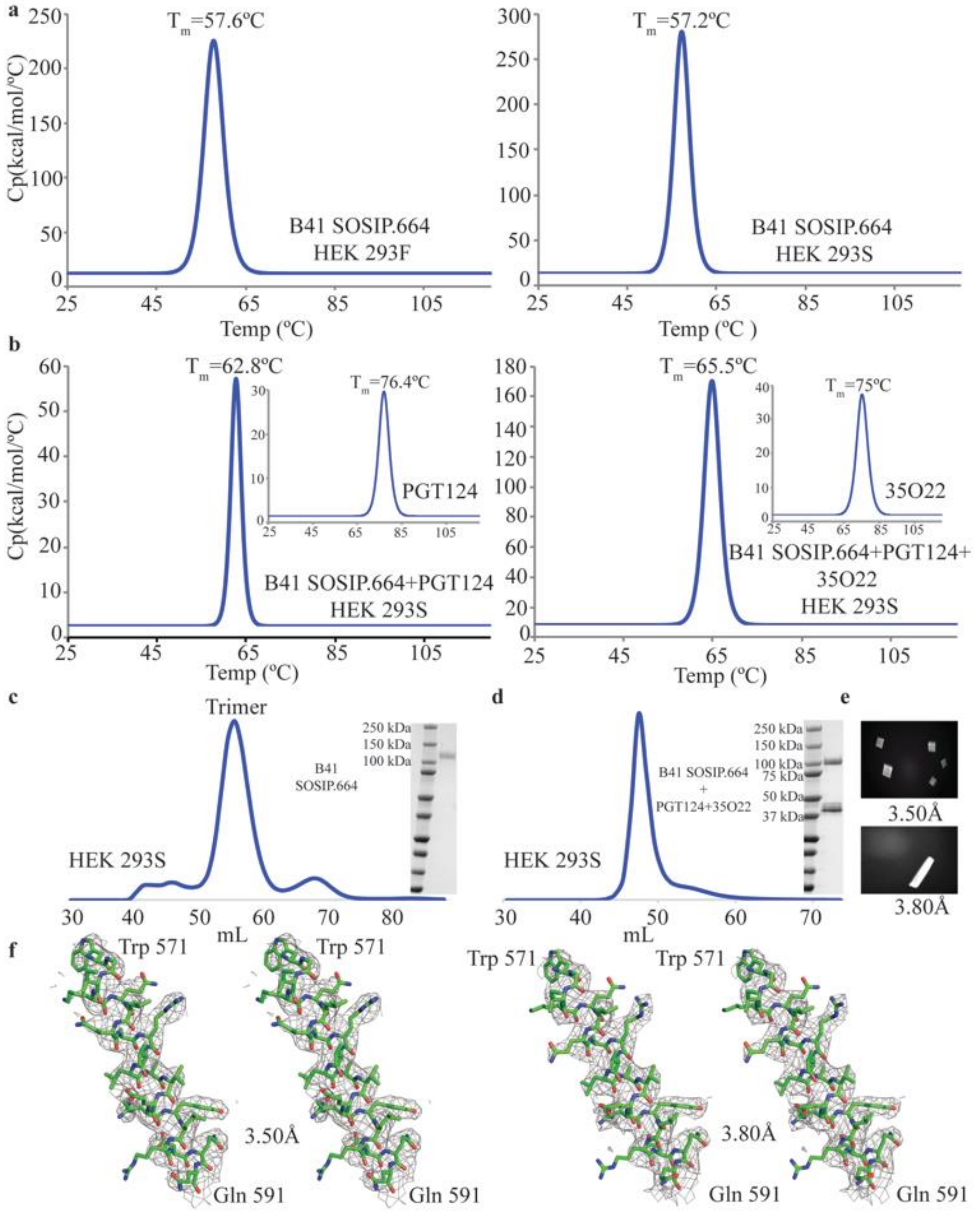

Supplementary Fig. 1. B41 SOSIP.664 trimer thermostability, expression and crystallization. a Thermostability of B41 trimers produced in HEK 293S or HEK293F cells. **b**

Enhanced thermostability and stabilization of B41 after binding Fabs PGT124 and 35O22. The insets show thermostability of unbound Fabs PGT124 and 35O22. **c** SEC 16/600 column profiles of B41 Env proteins produced in HEK 293S cells after 2G12 affinity purification, together with non-reducing SDS-PAGE analysis (inset). **d** SEC profile of the complex of B41 Env with PGT124 and 35O22 (left) was confirmed using a non-reducing SDS-PAGE gel (right). **e** Crystals of the complex of B41 SOSIP.664 trimers bound to PGT124 and 35O22 were obtained under two different conditions (see main text) and diffracted to 3.5 Å and 3.8 Å resolution. **f** A stereo representation of the *2Fo-Fc* electron density map at 1.0σ for a section of the HR1c region of the B41 SOSIP.664 complex.

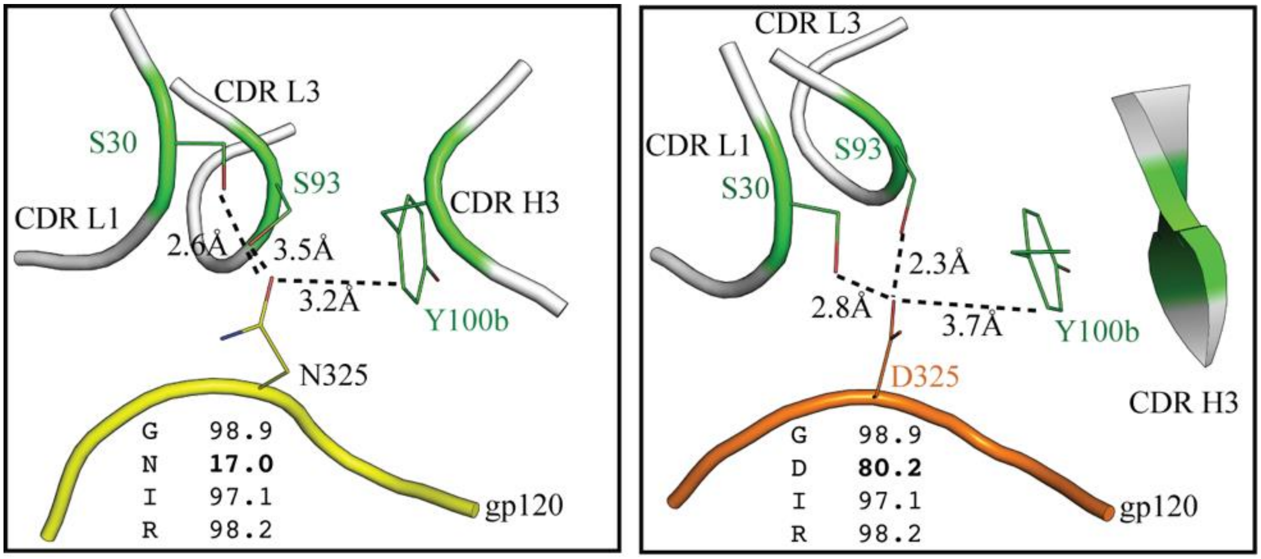

Supplementary Fig. 2. Interaction between PGT124 and the base of the gp120 V3 region. The interaction between the PGT124 CDR loops and N325 of the GNIR motif at the base of the gp120 V3 region of the B41 SOSIP.664 trimer is shown in the left panel. The interactions are indicated with dotted lines. For comparison, the right panel shows the interaction between the PGT124 CDR loops and D325 of the GDIR motif at the base of the JRCSF gp120 V3 region (PDB ID: 4R2G). The conservation (frequency, in percent) of each amino-acid residue of the G(D/N)IR motif among 5,451 aligned HIV-1 sequences is shown at the bottom of each panel; D325 is much more prevalent than N325.

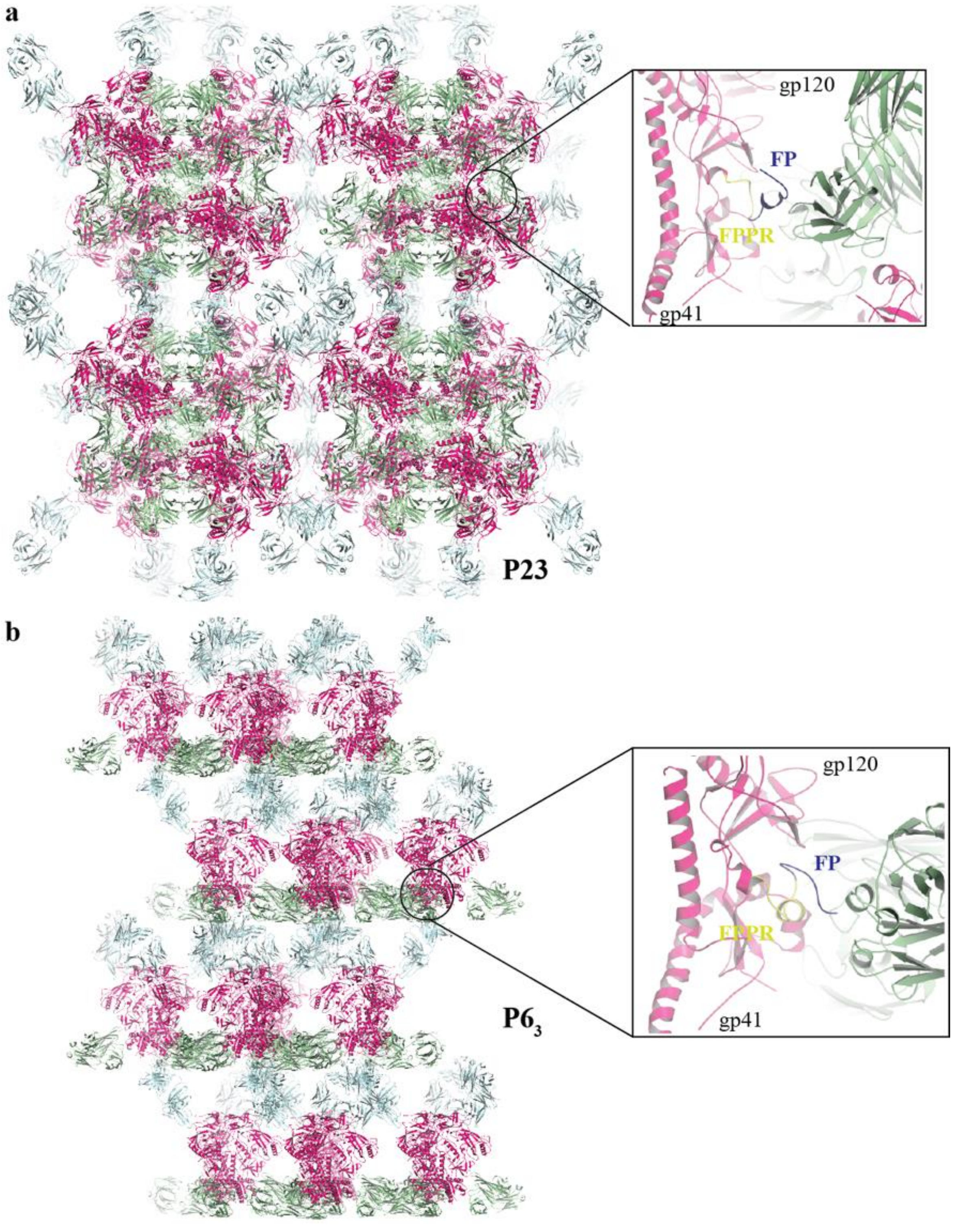

Supplementary Fig. 3. B41 SOSIP.664 trimer crystal packing. a Cartoon representation of the packing of the B41 SOSIP.664 trimer (pink) bound to Fabs PGT124 (cyan) and 35O22 (green) in

the cubic crystal (P23) at 3.50 Å. **b** The same representation as in (a), but for the hexagonal crystal ($P6_3$) at 3.80 Å. The insets show the crystal contacts of FP (blue) and FPPR (yellow) with 35O22 from a symmetry mate.

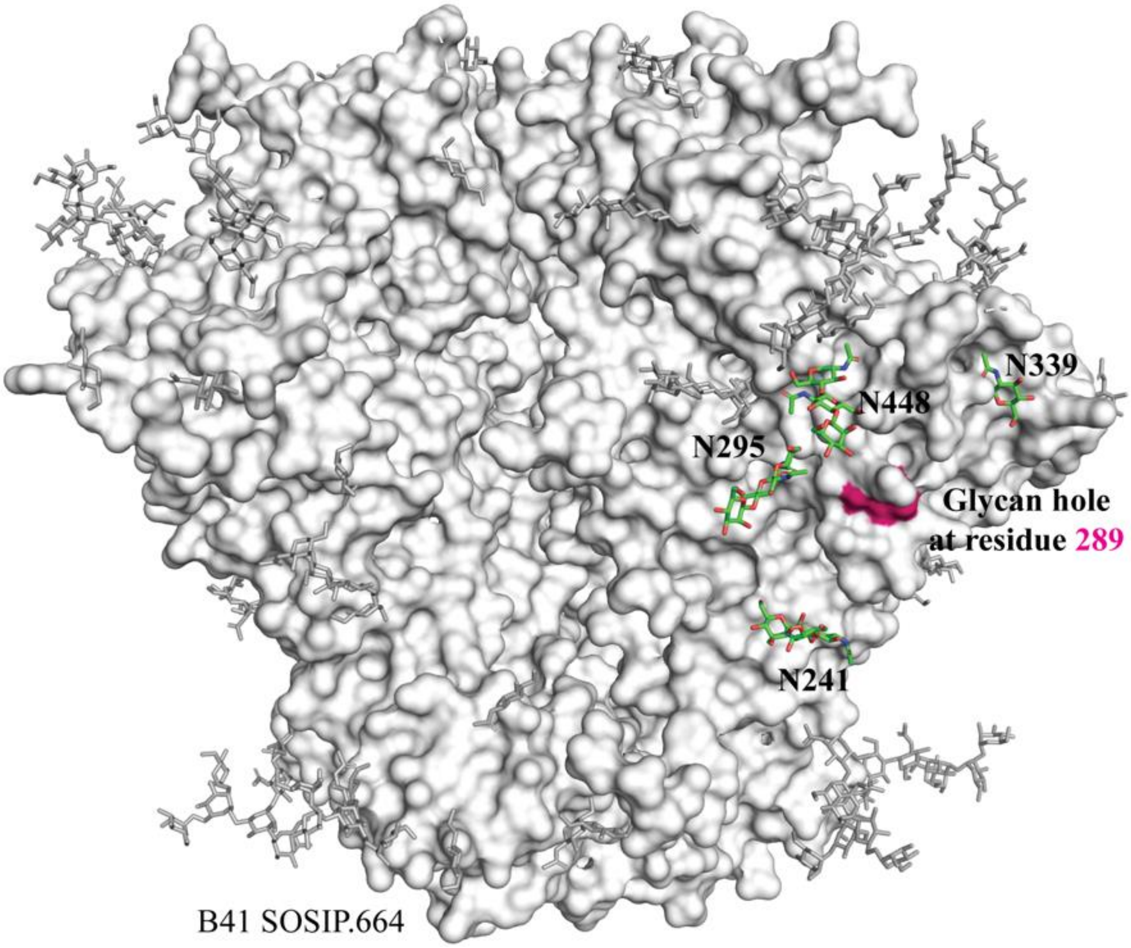

Supplementary Fig. 4. Glycan hole in B41 SOSIP.664 trimer. A glycan hole created by the absence of a glycan at residue 289 (pink). Glycans present at neighboring positions 241, 295, 339 and 448 (green) are depicted and labeled**.** Other glycans observed in the structure are shown as stick representations (light grey).

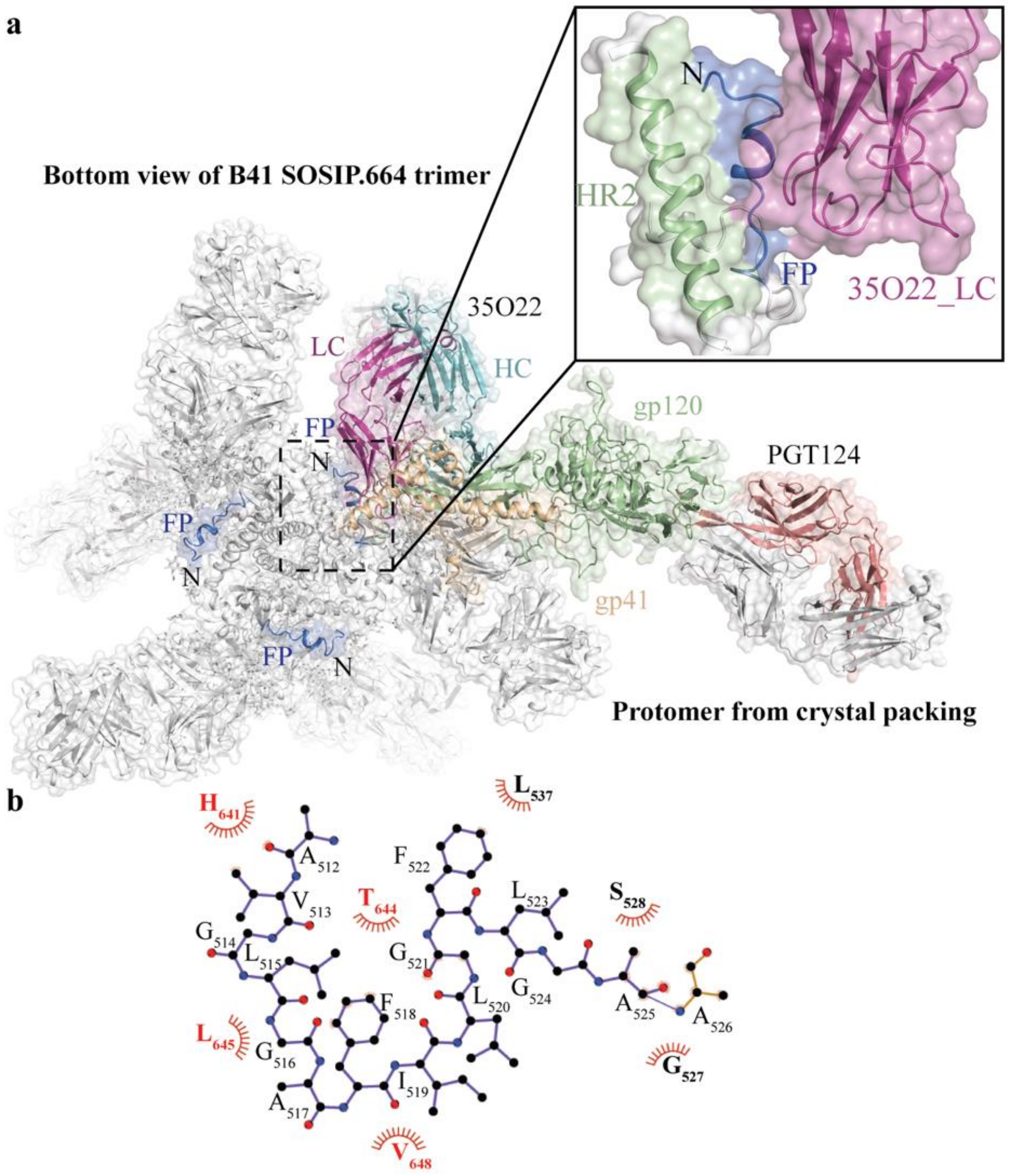

Supplementary Fig. 5. Packing of the fusion peptide in the crystal. a Bottom view of the B41 SOSIP.664 trimer (grey) shown in cartoon representation, overlaid with Fabs PGT124 and 35O22 in a transparent surface illustrating the location of the free N-terminal FP (blue). The Env protomer (gp120:green, gp41:orange) bound to Fabs PGT124 (light chain, LC: grey, heavy

chain, HC: light pink) and 35O22 (LC: pink, HC: cyan) in the crystal lattice is shown. The top inset highlights the FP conformation that is sandwiched between the light chain of 35O22 (pink) and the gp41 HR2 (green) from a neighboring protomer in the trimer. **b** Ligplot tool indicates the hydrophobic interactions between the FP (in stick format, with individual residues labeled) and HR2 residues (bold red color) from a neighboring protomer.

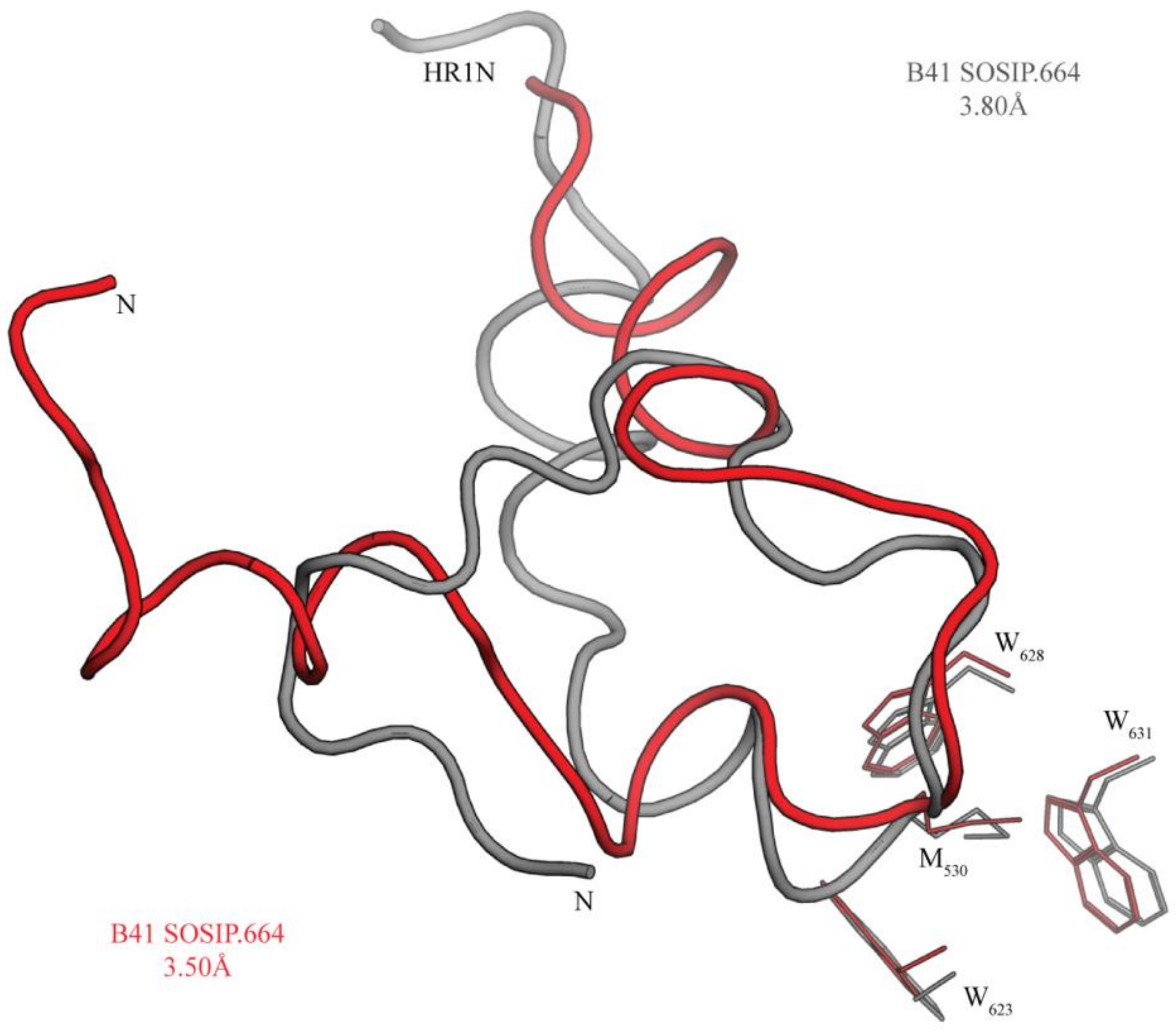

Supplementary Fig. 6. M530 is an anchor point for the different conformations of the FP. Overlay of the polypeptide backbone (represrented as a tube) of FP, FPPR, and HR1N of the B41 SOSIP.664 trimer at 3.5 Å (red) and 3.8 Å (grey), illustrating how M530 remains embedded in the tryptophan clasp (W623, W628, W631) and acts as an anchor despite the conformational variation observed in the FP and FPPR.

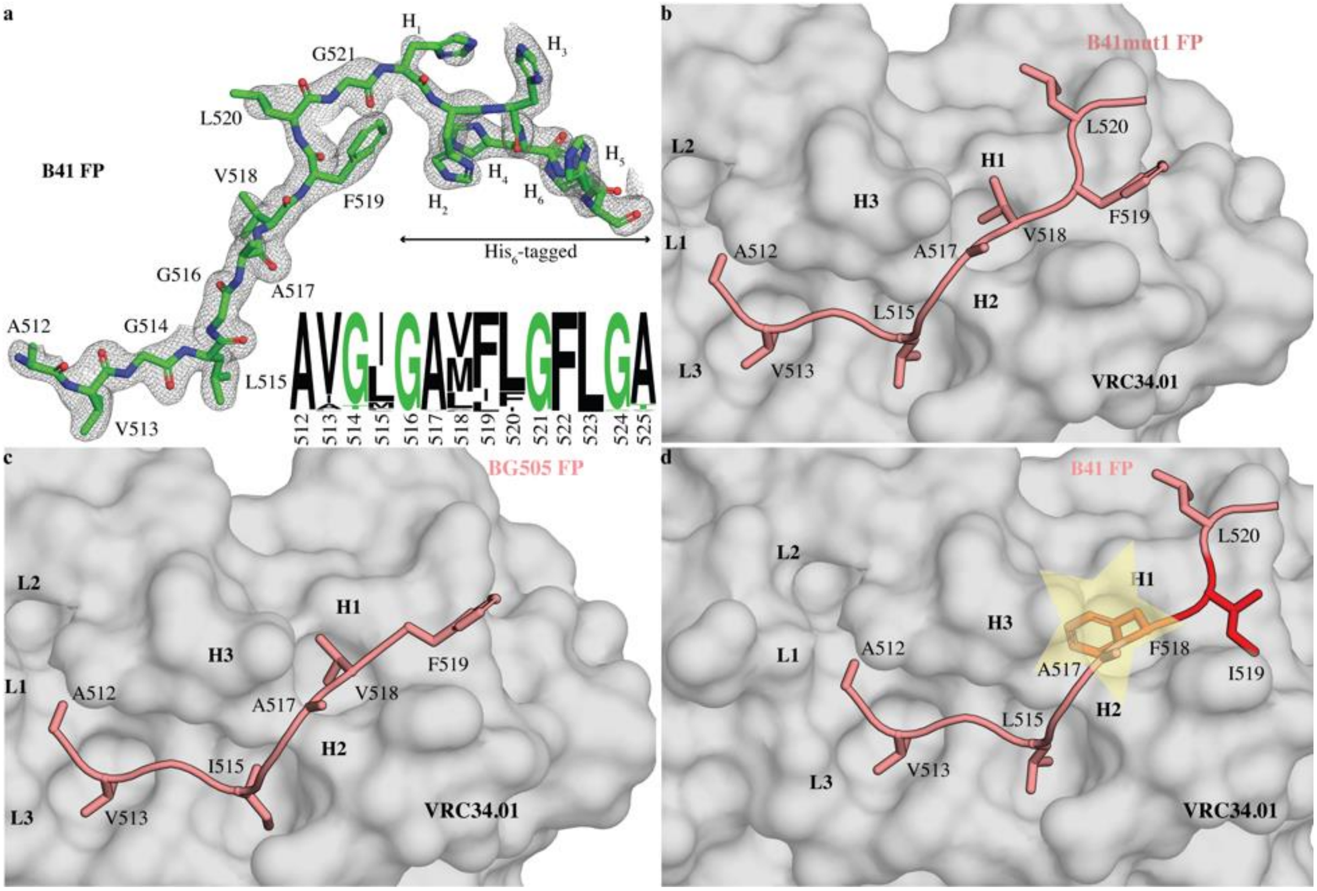

Supplementary Fig. 7. Crystal structure of the optimized FP of B41 and comparison with BG505. a 2Fo-Fc electron density omit map (1.0σ) of the optimized His-tagged fusion peptide of B41 with the final FP structure shown in stick representation. The Weblogo tool is used to illustrate the conservation and frequency of each amino-acid residue of the fusion peptide among 5,451 aligned HIV-1 sequences [1]. **b** Crystal structure of B41mut1FP (shown as a backbone tube with side chains in sticks; salmon color) bound to VRC34.01. The location of the CDRs from the light and heavy chains are shown in bold. **c** A corresponding representation of the BG505 FP bound to VRC34.01, derived from its crystal structure (PDB 5I8H). **d** Residues F518 and I519 (shown in red) of the wild-type B41 FP are modeled on the crystal structure of the B41mut1FP mutant in complex with VRC34.01. A clash is predicted between FP F518 and VRC34.01, as shown by the light yellow star.

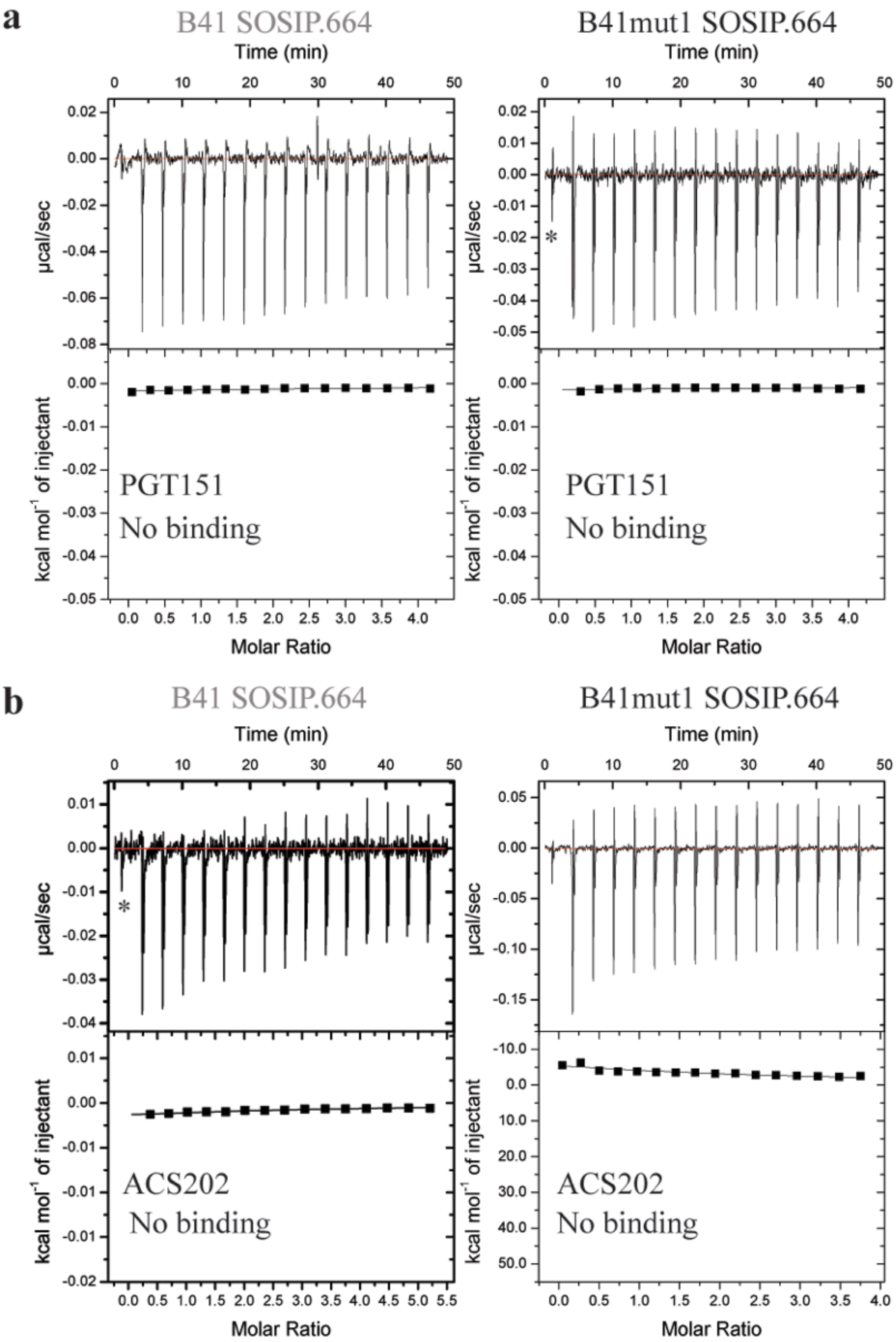

Supplementary Fig. 8. Biophysical characterization of bnAb binding to the B41 SOSIP.664 trimer and FP-optimized B41mut1 SOSIP.664 trimer. In an ITC assay, there was no

detectable binding of either trimer to (**a**) PGT151 or (**b**) ACS202. Both trimers were produced in HEK293S cells.

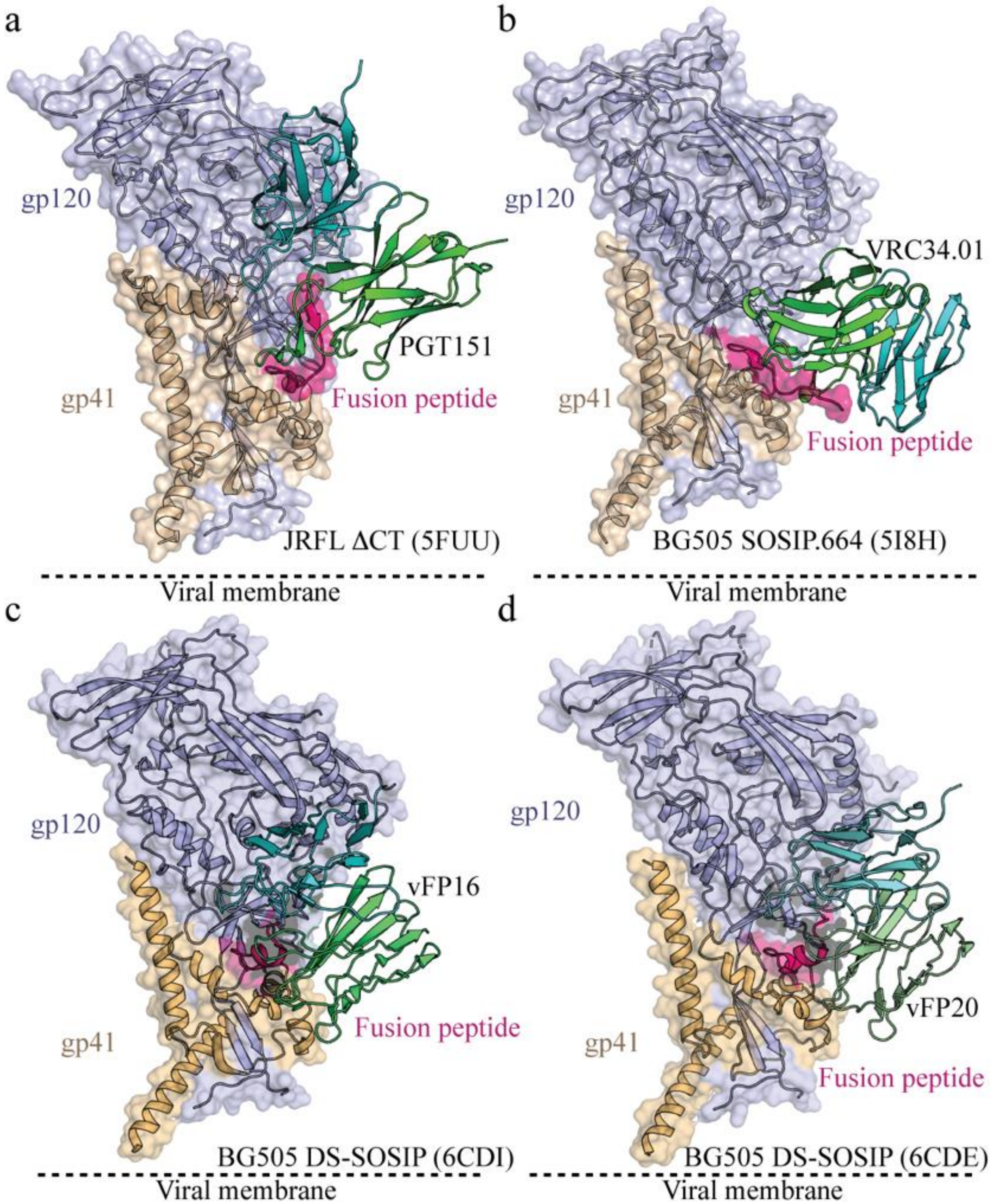

Supplementary Fig. 9. Comparison of bNAb angle of approach to the fusion peptide. a-b PGT151 approaches the FP of WT JRFL ΔCT from a more vertical angle (a), whereas VRC34.01 interacts with the FP of BG505 SOSIP.664 via an approach that is more parallel to the viral membrane (b). **c-d** The vFP16 and vFP20 FP-directed bNAbs both interact similarly

with the FP of the BG505 DS-SOSIP trimer, but do so differently from the FP interaction with PGT151 or VRC34.01.

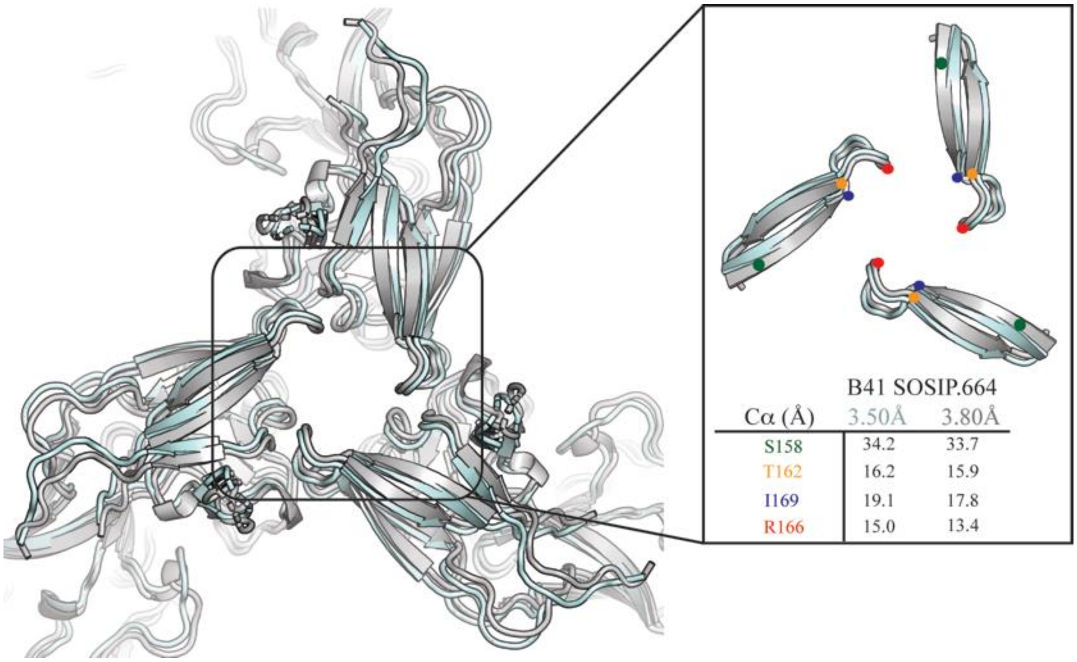

Supplementary Fig. 10. HIV Env opening at apex. Overlay of the B41 SOSIP.664 trimer at 3.50 Å (cyan) and 3.80 Å (grey) resolutions. Inset shows overlay of V2 loops between the two structures and inter-V2 (Cα) measurements at the interior (S158 in green), base (T162 in orange and I169 in blue) and tip (R166 in cyan) of the loop that suggests some breathing.

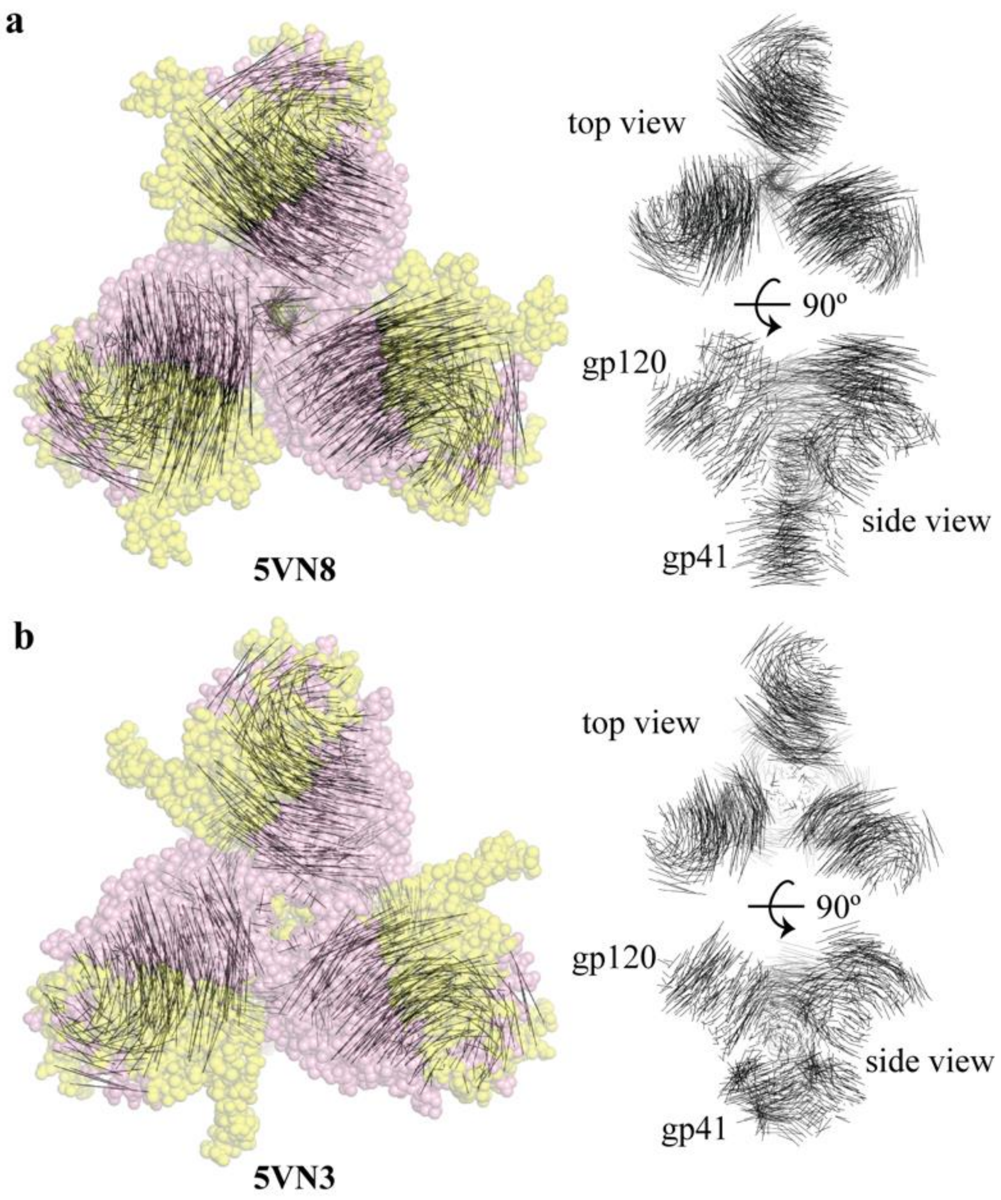

Supplementary Fig.11. Structural movement between open and closed B41 SOSIP.664 structures. a-b Overlay of aligned open (5VN8 and 5VN3, yellow) and closed B41 SOSIP.664 (pink) trimers (left panels), with the distances between each Cα position on trimer shown in black lines. The two ends of each line represent the positions of the same residue in the two structures. The right panels are top and side views that trace the varying degrees of movement (black lines) between the aligned open and closed structures for both the gp120 and gp41 subunits of the Env trimer.

Supplementary Table 1. X-ray crystallographic data and refinement statistics for the B41 SOSIP.664 trimer complex with Fabs PGT124 and 35022.

**Data collection**	**B41 SOSIP.664 + Fab PGT124 + Fab 35O22**	
Beamline	APS 23-ID D	SSRL 12-2
Wavelength (Å)	1.03317	0.97946
Detector	Pilatus	Pilatus
Space group	P23	$P6_3$
Cell dimensions a, b, c (Å)	212.3, 212.3, 212.3	129.3, 129.3, 313.0
Resolution (Å)	50.0-3.5 (3.63-3.50)[a]	50.0-3.8 (3.94-3.80)[a]
Total reflections	1,485,491	313,463
Unique reflections	39,580	28,747
Redundancy	37.5 (30.6)[a]	10.9 (9.6)[a]
Completeness (%)	100 (100)[a]	99.9 (99.9)[a]
$<I/\sigma_i>$	10.1 (1.0)[a]	6.4 (1.8)[a]
R_{sym}[b]	0.62 (>1.0)[a]	0.32 (1.0)[a]
R_{pim}[c]	0.10 (0.84)[a]	0.09 (0.32)[a]
$CC_{1/2}$[d]	84.4 (39.2)[a]	77.1 (27.6)[a]
**Refinement statistics**		
Resolution (Å)	50.0-3.5	41.9-3.8
Reflections (work)	37,512 (2668)[a]	27,362 (2680)[a]
Reflections (test)	1951 (127)[a]	1359 (133)[a]
R_{cryst}[e] / R_{free}[f] (%)	30.4 / 32.1	29.4 / 31.1
Wilson B-value (Å^2)	106	99
Average B-value (Å^2)	126	125
gp120/gp41	110/86	112/91
PGT124 (V_H/V_L)	144/134	129/120
PGT124 (C_H1/C_L)	188/184	139/112
35O22 (V_H/V_L)	77/83	110/115
35O22 (C_H1/C_L)	177/155	218/206
**RMSD from ideal geometry**		
Bond length (Å)	0.005	0.004
Bond angles (°)	0.84	0.91
**Ramachandran statistics (%)**[g]		
Favored	93.02	93.17
Allowed	6.57	6.76
Outliers	0.41	0.07
**PDB ID**	6MCO	6MDT

[a]Numbers in parentheses are for highest resolution shell
[b]$R_{sym} = \Sigma_{hkl} \Sigma_i | I_{hkl,i} - <I_{hkl}> | / \Sigma_{hkl} \Sigma_i I_{hkl,i}$, where $I_{hkl,i}$ is the scaled intensity of the ith measurement of reflection h, k, l, and $< I_{hkl}>$ is the average intensity for that reflection
[c]$R_{pim} = \Sigma_{hkl} (1/(n-1))1/2 \Sigma_i | I_{hkl,i} - <I_{hkl}> | / \Sigma_{hkl} \Sigma_i I_{hkl,i}$, where n is the redundancy
[d]$CC_{1/2}$ = Pearson Correlation Coefficient between two random half datasets

[e]$R_{cryst} = \Sigma_{hkl} | Fo - Fc | / \Sigma_{hkl} | Fo |$ x 100

[f]R_{free} was calculated as for R_{cryst}, but on a test set comprising 5% of the data excluded from refinement

[g]These values were calculated using MolProbity [2]

Supplementary Table 2. X-ray crystallographic data and refinement statistics for the B41 mut1FP complex with VRC34.01.

**Data collection**	**B41 mut1FP + Fab VRC34.01**
Beamline	APS 23-ID B
Wavelength (Å)	1.0332
Detector	Eiger16M
Space group	$P2_1$
Cell dimensions a, b, c (Å), °	42.4, 123.6, 101.2, β=90.8
Resolution (Å)	50.00-1.97 (2.01-1.97) [a]
Total reflections	230,268
Unique reflections	72,903
Redundancy	3.2 (3.1) [a]
Completeness (%)	99.3 (99.6) [a]
$<I/\sigma_i>$	11.4 (1.1) [a]
R_{sym} [b]	0.10 (0.9) [a]
R_{pim} [c]	0.05 (0.4) [a]
$CC_{1/2}$ [d]	89.0 (64.0) [a]
**Refinement statistics**	
Resolution (Å)	38.1-1.97
Reflections (work)	69,184 (2183) [a]
Reflections (test)	3665 (135) [a]
R_{cryst} [e] / R_{free} [f] (%)	19.9 / 23.8
Protein atoms	6796
Water molecules	572
Wilson B-value ($Å^2$)	29
Average B-value ($Å^2$)	38
VRC34.01/B41 mut1FP	38/42
Waters	40
**RMSD from ideal geometry**	
Bond length (Å)	0.003
Bond angles (°)	0.61
**Ramachandran statistics (%)** [g]	
Favored	96.49
Allowed	3.51
Outliers	0.00
**PDB ID**	6ME1

[a]Numbers in parentheses are for highest resolution shell

[b]$R_{sym} = \Sigma_{hkl} \Sigma_i | I_{hkl,i} - <I_{hkl}> | / \Sigma_{hkl} \Sigma_i I_{hkl,i}$, where $I_{hkl,i}$ is the scaled intensity of the ith measurement of reflection h, k, l, and $< I_{hkl}>$ is the average intensity for that reflection

[c]$R_{pim} = \Sigma_{hkl} (1/(n-1))1/2 \Sigma_i | I_{hkl,i} - <I_{hkl}> | / \Sigma_{hkl} \Sigma_i I_{hkl,i}$, where n is the redundancy

[d]$CC_{1/2}$ = Pearson Correlation Coefficient between two random half datasets

[e]$R_{cryst} = \Sigma_{hkl} | Fo - Fc | / \Sigma_{hkl} | Fo | \times 100$

[f]R_{free} was calculated as for R_{cryst}, but on a test set comprising 5% of the data excluded from refinement
[g]These values were calculated using MolProbity [2]

References:

1. Waterhouse AM, Procter JB, Martin DM, Clamp M, Barton GJ. Jalview Version 2--a multiple sequence alignment editor and analysis workbench. *Bioinformatics* **25**, 1189-1191 (2009).

2. Chen VB, *et al.* MolProbity: all-atom structure validation for macromolecular crystallography. *Acta Crystallogr D Biol Crystallogr* **66**, 12-21 (2010).
