# Supplementary material for: Capturing the inherent structural dynamics of the HIV-1 envelope glycoprotein fusion peptide
Source: Nat Commun. 2019 Feb 15;10:763. doi: 10.1038/s41467-019-08738-5 (PMC6377653; doi:10.1038/s41467-019-08738-5)
Supplement: Supplementary file 3 — Description of Additional Supplementary Files [file 41467_2019_8738_MOESM3_ESM.pdf]

**Description of Additional Supplementary Files**

**File Name:** Supplementary Movie 1.

**Description: Env trimer twisting.** The B41 SOSIP.664 trimers from the 3.5Å (red) and 3.8Å (grey) resolution structures were aligned on their gp41 Cα coordinates to reveal twisting of the gp120 subunits relative to each other. Alignment and video rendering were done using PyMol [1].

**File Name:** Supplementary Movie 2.

**Description: Protomer sub-domain movement.** Alignment of the gp41 domains of B41 SOSIP.664 Env trimer at 3.5Å (red) and 3.8Å (grey) on their Cα coordinates show gp120 and gp41 domain movements. The gp120 sub-domain moves in an anticlockwise direction. Alignment and video rendering were done using PyMol [1].

**File Name:** Supplementary Movie 3.

**Description: CD4-induced opening of trimer**. Morph between the Cα coordinates of the open CD4/17b-bound and the closed PGT124/35O22-bound pre-fusion B41 SOSIP.664 Env trimer highlights the large movement of both the gp120 and the gp41 sub-domains. Morphing and video rendering were done using PyMol [1].

**References:**

1. Schrodinger L. The PyMOL Molecular Graphics System, Version 1.8. (2015).
